# Everything you wanted to ask about EEG but were afraid to get the right answer

**DOI:** 10.1186/1753-4631-3-2

**Published:** 2009-05-26

**Authors:** Wlodzimierz Klonowski

**Affiliations:** 1 Institute of Biocybernetics and Biomedical Engineering, Polish Academy of Sciences, Warsaw, Poland

## Abstract

We answer several important questions concerning EEG. We also shortly discuss importance of nonlinear methods of contemporary physics in EEG analysis. Basic definitions and explanation of fundamental concepts may be found in my previous publications in *NBP*.

*It is a magnificent feeling to recognize the unity of complex phenomena which appear to be things quite apart from the direct visible truth*.

Albert Einstein

## Q1. Why anybody might be afraid of getting the right answers about EEG?

**A1.** Habit is a second nature. Before personal computers came into in medicine in 1980's EEG signals were registered on a wide paper tape When EEG was registered on a paper tape vertical lines 3 cm away, moving 1.5, 3.0, or 6.0 cm/sec depending on the system, doctor who interpreted this EEG could easily observe frequency of EEG waves by counting number of pen sways (wave ridges) in one second if there were, say, from 3 up to 30 wave maxima between two vertical lines. From this paper-tape period come classical names of EEG-bands, in particular *α *and *β *bands; if there were waves of small frequency they could be too flat to be observed by naked eye, while those with frequencies higher than 30 merged together due to the width of pen's line and were considered to be just a noise. So, when development of personal computers enabled numerical registration of EEG-signals and their spectral analysis using linear methods like FFT developed at the same time, Medical Doctors accepted those methods quite easily and even the new 'slow' and 'quick' brainwaves bands were introduced. It does not matter that EEG-signals are resultant of activity of many brain cells – the existence of a particular frequency in the FFT does not necessarily mean that there is an oscillator in the brain at that frequency. **New nonlinear methods **should be much more appropriate for EEG-analysis but are still used only in research and not in everyday clinical practice because they **are not yet rooted in medical tradition and they meet strong barrier of doctors' habits**. Somebody who got accustomed to spectral methods does not want to learn new methods not even to ask questions the right answers to which could make the person to understand and admit that the methods he/she has been using might be often wrong or at least misleading. Basic definitions and explanation of fundamental concepts may be found in many papers and books [1,2].

## Q2. Is brain deterministic or stochastic?

**A2.** Human brain may neither act as a fully deterministic system because it would not be able to invent anything new, nor may it act as a fully stochastic system because it would not be able to learn and exactly repeat any sequence of thoughts. The answer to this dilemma lies in nonlinear dynamics and deterministic chaos – **human brain is a complex nonlinear system showing complicated emergent properties, including consciousness**. It is much more appropriate to use nonlinear methods for analysis of signals generated by such a complex nonlinear system, despite the fact that for short time intervals linear methods like FFT may work o.k. Nonlinear methods may be applied to linear signals – one might try to approximate straight line using parabolic function only to find that coefficient of the quadratic term was practically equal zero. It is the opposite that obviously fails – if one uses linear approximation then one will never be able to appropriately approximate a nonlinear function on a sufficiently long interval, but on a short interval linear predictability may work o.k. It is unbelievable but in the XXI century some scientists still maintain they have demonstrated by using methods like linear forecasting or surrogate data tests of EEG time series that EEG is linear signal as if the human brain is a linear system. Although linear systems may have a small range of applicability, it is inappropriate to use a linear system to deal with the highly nonlinear complexity of the brain. Perfect linear system is a kind of abstraction like an ideal gas.

## Q3. Is EEG a linear signal?

**A3.** No, EEG-signal generated by brain **is nonlinear**. While practically all researchers do agree that human brain is the most complex system we know, many researchers still claim that brain is linear, or at least that EEG-signals generated by brain are linear, without realizing that these 'believes' contradict one another. Many biomedical researchers are **'infected with Human Linearity Virus' (HLV) **– they 'think linearly' and ignore the facts that human body, and, in particular, human brain are complex nonlinear systems generating nonstationary nonlinear signals, and that appropriate analysis of such signals does need new nonlinear methods. We have demonstrated an example of astonishing similarity between economic crisis and epileptic seizure in the brain if appropriate nonlinear methods (like Higuchi's fractal dimension) are applied to the analysis of a 'signal' generated by an 'economic organism' – time series of Dow Jones index during the period of 'big crash' – and EEG-signal during epileptic seizure [1,3].

## Q4. Is EEG a stationary signal?

**A4.** No, EEG signal **is nonstationary**. In general, biosignals are ***'3N' – Nonstationary*, *Nonlinear*, *Noisy***. *Nonstationarity *means that signal's statistical characteristics change with time. The brain activity is essentially nonstationary. Quasi-stationary segments in EEG have duration about 0.25 sec [4]. The basic source of the observed nonstationarity in EEG signal is not due to the casual influences of the external stimuli on the brain mechanisms, but rather it is a reflection of switching of the inherent metastable states of neural assemblies during brain functioning. EEG-signal recorded from a scalp electrode is influenced by different deeper brain structures, each 'transmitting' with different and changeable intensity; so, in a fraction of a second the main source of the registered signal often moves from one brain structures to another. And if source of a signal changes with time then the signal is obviously nonstationary. Nonstationarity arises also because of different time scales involved in the dynamical process – dynamical parameters are sensitive to the time scales and hence in the study of brain one must identify all relevant time scales involved in the process to get an insight in the working of brain [5]. It is extremely important that fractal methods easily detect nonstationarities in the analyzed signals, nonstationarities that are not easily detectable by linear methods like FFT [1]. Nonstationarities in EEG are also due to pathological changes, for example epileptic seizures, or to changes of the physiological state, for example passing from one sleep stage to another.

## Q5. Is it necessary to apply surrogate data method to EEG time series?

**A5.** Surrogate data tests may demonstrate that it is not impossible that the analyzed signal is generated by a linear Gaussian process, but such tests may never prove that this is really the case. On the other hand, it is obvious that signals generated by human brain, the most complex system we know, are really complex nonlinear signals and that **there exist much more general premises to assume that EEG-signals are nonlinear **(cf. A2. above) and linear methods may eventually be used only for sufficiently short time-scale. For example, if an organism is placed under the influence of ionizing radiation for a short period i.e. if only a small dose of radiation is absorbed the effect exerted on the organism may be positive, proportional to the absorbed dose if that was sufficiently small, while for longer periods of exposition and higher absorbed doses the effect becomes strongly negative and strongly nonlinear, leading to the death of the organism; this phenomenon is called *radiation hormesis *[1,6]. When analyzing systems that show hormetic effects we clearly have to look for nonlinear dose-effect relation. Similarly, even before we were able to have a look onto the Earth from cosmic space to affirm that it really has spherical shape there were enough of other premises to affirm the assumption of Earth's spherical shape. Even if a geodesist had measured many triangles on the Earth surface and found that the sum of angles in each of those triangles was not statistically different from 180° (that is of course true for triangles which sides are short in comparison with Earth's radius) nobody would have maintained that it was the evidence that the Earth is flat. What seems to be true on comparatively short scale (in time or in space) may no longer be true on longer scale (cf. A3. above) – a neuroscientists who maintains that EEG is linear is like a geodesists who maintains that Earth is flat.

## Q6. Is the routine EEG-acquisition done properly?

**A6. No, routine EEG-acquisition is done improperly from the point of view of neuroscientific research**. It is obvious that the higher is the frequency of a wave the more information it may carry, but frequencies exceeding roughly 70–90 Hz are filtered out by EEG-data acquisition systems hardware, and so are very low frequencies smaller than 0.5 Hz. It is so due to paper tape tradition and doctors' habits (cf. A1. above). On the other hand, neuroscientists have found that some tasks, like e.g. face perception, elicit frequencies up to 250 Hz when the stimuli are processed by human brains [7]. Most of manufacturers of EEG apparata do not leave any possibility for users to register unfiltered (raw) EEG-signal for further analysis with new analytical methods. Only recently some manufacturers like NeuroConn GmbH (Ilmenau, Germany) supply EEG-systems that enable registration of unfiltered signals.

## Q7. What is 'normal EEG'?

**A7.** In Medicine two plus two not always equals four and that is why there is no alternative to Personalized Medicine provided by well trained Medical Doctors. The normal body temperature 36.6°C is the norm, but it is not easy to point to any other norm like that. It is **erroneous belief that in Medicine the normal value equals the population average value **– so called 'normative databases' of EEG, no matter how large, do not give a possibility of 'reliable comparison' to decide if the given case is 'normal' or 'abnormal'. Moreover, those databases are mostly based on spectral linear analysis of biosignals like EEG. Human organism (and human brain in particular) is a highly complex nonlinear systems, and that is why standardized approach based on 'stiff' protocols may lead to serious errors of judgment. There are differences in defining 'normal' ranges even between quite reliable sources. For example, according to NLM 'Normally, the ICP [Intracranial Pressure] ranges from 1 to 15 mm Hg' [8] but other sources give ranges like 8 to 18 mm Hg; anyway, what for one person is a quite high ICP for another may be quite low. Similarly, **nothing like 'normal EEG' exists**, one may only compare EEG of the same person in different time periods, in particular 'before' and 'after', e.g. comparing EEG before and after administration of different drugs. Markers supplied by Biomedical Physicists, e.g. quantitative descriptors of EEG-signal adapted from Nonlinear Dynamics, may help in better assessment of various spontaneous or evoked, normal and pathological functional states of the brain in neuropsychiatric patients, and so may be helpful in deciding diagnosis, treatment, and prognosis.

## Q8. Is it possible to model EEG-signals with systems of ordinary differentials equations (ODE)?

**A8.** Using systems of ODE with several parameters that may not exactly be calculated from carefully done experiments one may modelled practically any process. For example, Liley's model consists of 14 first order nonlinear ODE with 29 physiological and anatomical parameters, some of which may take values from continuous ranges of possible values [9]. Using numerical computer methods one may solve such systems of ODE which might as well model 'problems' like **'influence of the baldness of the forward basketball players on the results of NBL matches'**. One may even predict results of several consecutive matches based on such a model. To characterize baldness one needs several parameters, plus one needs more obvious but not exactly measurable parameters characterizing momentary physical fitness of all players, etc. There exist infinite number of combinations of ODE parameters that will lead to the same prediction on some short interval, but will differ tremendously on a longer time interval. So, such models do not explain practically anything nor they give a possibility to measure some parameters of the system under consideration. Even a model consisting of only 3 simple quasilinear ODE with 3 parameters (so called Lorenz equations, [1]) of which 2 are constant while 1 slightly changes may lead to quite unexpected behaviour. These remarks concern not only modelling of EEG-signals but practically any ODE model with several parameters that should be found experimentally, like ECG-signals, membrane transport, sugar level regulation, etc, etc.

## Q9. What are shortcomings of linear methods?

**Figure 1:**
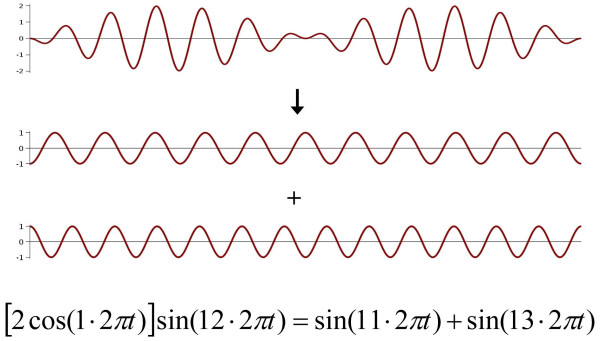
Fourier decomposition of a signal of frequency 12 Hz with the amplitude modulated with frequency 1 Hz (upper) results in  two harmonic signals  – one with frequency 11 Hz and another with frequency 13; the basic frequency 12 Hz completely disappears.

**A9. Linear methods **like FFT (Fast Fourier Transform), WT (wavelet transform), or MP (Matching Pursuit) **work properly only for stationary signals but assumptions of stationarity required for the correct use of these algorithms are often ignored**. WT and MP has better accuracy than FFT but much bigger ambiguity in signal decomposition. Linear methods may lead to very misleading results. E.g. if in a measured signal one observes regular waves of frequency 12 Hz with amplitude modulated with frequency 1 Hz, then Fourier decomposition of this signal leads to two components, each of amplitude equal half of that of the analyzed signal, with frequencies 11 Hz and 13 Hz respectively, while the basic frequency of the analyzed signal (12 Hz) does not appear at all in the Fourier spectrum; such a result may be predicted even without any calculations, from a simple trigonometric formula that was used in high schools in 'pre-computer era' for transforming a sum of two sine functions into a product of sine and cosine that could be easily computed with a slide (logarithmic) rule (Figure 1). We do need new nonlinear methods of biosignal analysis; otherwise while living in XXI century, we will still be plunged in XIX century 'linear science' of Fourier and Markov.

## Q10. What are advantages of nonlinear methods?

**Figure 2:**
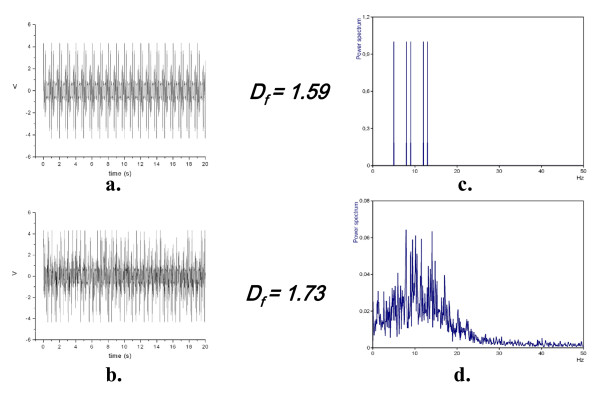
**FFT applied to two similar – stationary (upper left, a.) and nonstationary (lower left, b.) signals gives dramatically different results (right, c. and d.)**, while application of Higuchi's algorithm gives quite similar values of average fractal dimension, *D*_*f*_, of both signals. The stationary signal was composed of five harmonic waves of different frequencies, and then randomly chosen small segments were removed from that stationary signal, so forming a nonstationary signal that when decomposed using Fourier leads to a very 'rich' spectrum of frequencies. Routine artefacts’ correction in EEG-signals may lead to similar unreliable cases.

**A10.** One may apply methods of nonlinear analysis, for example Higuchi's fractal dimension method, *D*_*f *_[1,10], to any signal. *D*_*f*_'s value, always between 1.0 and 2.0, is just a measure of what is called *signal's complexity*. Unlike fractal dimension in phase space [1]*D*_*f *_is calculated directly in time domain; running fractal dimension, *D*_*f *_*(t)*, may be calculated using a moving window as short as 70–100 data points. It is not necessary to made surrogate data test before applying Higuchi's fractal dimension method because **it does not matter if the analyzed signal itself is 'really chaotic' **– it may be deterministic, stochastic, nonstationary and noisy. Moreover, generation of surrogate data often applies linear transformations like FFT and reverse FFT that may show serious shortcomings. E.g. FFT applied to similar stationary and nonstationary signals gives dramatically different results, while application of Higuchi's algorithm gives quite similar values of average fractal dimension, *D*_*f*_, of both signals (Figure 2). *D*_*f *_is linearly related to so called Hurst exponent that is a measure of the tendency for time series values to persist or to alternate and works on prediction of chaotic time series suggest that humans are sensitive to the Hurst exponent [11], and so must be sensitive also to *D*_*f*_.

## Conclusion

We all become more and more specialized in very narrow disciplines and we often do not know that the methods we want to apply in our research have been used for a long time in other disciplines. When we learn about it we are often amazed like Molier's Mr. Jourdain (*Le Bourgeois Gentilhomme *II. iv) who says: 'Good heaven! For more than forty years I have been speaking prose without knowing it'. Our philosophy is that to be applicable a method should preferably be really simple and easily understandable to non-specialists in the field. Some nonlinear methods like Higuchi's fractal dimension method are very simple – they draw from multiple disciplines and have multidisciplinary applications.
